# Smartphone Use and Sleep Quality in Chinese College Students: A Preliminary Study

**DOI:** 10.3389/fpsyt.2020.00352

**Published:** 2020-05-06

**Authors:** Qiuping Huang, Ying Li, Shucai Huang, Jing Qi, Tianli Shao, Xinxin Chen, Zhenjiang Liao, Shuhong Lin, Xiaojie Zhang, Yi Cai, Hongxian Chen

**Affiliations:** ^1^Department of Psychiatry, The Second Xiangya Hospital, Central South University, Changsha, China; ^2^Mental Health Institute of the Second Xiangya Hospital, Central South University, Chinese National Clinical Research Center on Mental Disorders (Xiangya), Chinese National Technology Institute on Mental Disorders, Hunan Key Laboratory of Psychiatry and Mental Health, Changsha, China; ^3^Center of Psychology Teaching and Research, Changsha Health Vocational College, Changsha, China; ^4^Department of Psychiatry, The Fourth People's Hospital of Wuhu, Wuhu, China; ^5^Department of Psychiatry, Brain Hospital of Hunan Province, Changsha, China; ^6^Department of Psychiatry, Geriatric and Somatic Diseases, Brain Hospital of Human Province, Hunan University of Chinese Medicine Clinical College, Hunan Mental Behavior Disorder Research Center, Changsha, China

**Keywords:** poor sleep quality, smartphone use, mobile phone addiction, college students, association

## Abstract

**Background:**

Chinese college students are at high risk of sleep problems, and smartphone use is common among this population. However, the relationship between smartphone use characteristics and sleep problems in Chinese college students has been inadequately studied. In this preliminary study, we examined the association of poor sleep quality with smartphone use in a sample of Chinese college students from a health vocational college in Changsha, China.

**Methods:**

A total of 439 college students completed a self-report questionnaire containing the Pittsburgh Sleep Quality Index (PSQI) and questions regarding demographic information, psychosocial factors, physical health, smartphone use characteristics, and mobile phone addiction (MPA).

**Results:**

The results showed that the prevalence of poor sleep quality (PSQI > 7) in Chinese college students was 9.8%. In multiple logistic regression analysis, poor sleep quality was significantly associated with male gender (OR: 2.80, P: 0.022), not having good physical health (OR: 2.61, P: 0.020), headache (OR: 2.47, P: 0.014), more severe depressive symptoms (OR: 2.17, P: 0.049), > four years of smartphone use (OR: 3.38, P: 0.001), > five hours of daily smartphone use (OR: 2.19, P: 0.049), and more severe inability to control MPA craving (OR: 2.04, P: 0.040).

**Conclusion:**

Our findings suggest that excessive smartphone use and MPA are associated with poor sleep quality in a sample of Chinese college students from a health vocational college. Because of the limited sample representativeness and cross-sectional design of this study, large-scale prospective representative studies are warranted to confirm these associations.

## Introduction

College students are at considerably high risk for sleep problems. Worldwide, the prevalence of insomnia in university students has been found to be 18.5%, much higher than the 7.4% rate in the general population (1). A cross-national survey reported an overall 10.4% prevalence of severe or extreme nocturnal sleeping problems in undergraduate university students during the previous month, with the lowest seen in Thailand (3.0%) and the highest in Indonesia (32.9%) (2). Chinese university students are also at increased risk of sleep problems; for example, 25.7% of university students have sleep problems, a rate two to three times that of the general Chinese population (7.4-15.0%) (1, 3, 4). Evidence from empirical studies has shown that sleep disturbance is a major risk factor for a variety of physical and mental morbidities and increased mortality, including obesity, cardiovascular diseases, depression, and cognitive impairment (5–10). For students, sleep disorder is also significantly associated with poor academic performance and elevated risk of suicidal behaviors (11, 12).

A diversity of factors have been reported to be associated with sleep problems in university students, such as smoking, academic failure, depression, anxiety, and stress (13–16). In recent years, because of the rapid development of communications and information technologies, smartphone use has been very popular in young people, particularly university students. While smartphone use has made our lives more convenient, it also has negative effects on the health of users. For example, in the international literature, sleep problems in university students and adolescents have been significantly associated with mobile phone addiction (MPA) and various measures of problematic smartphone use, including long duration of smartphone usage, late-night smartphone use, bedtime smartphone use, and excessive use of a smartphone (17–23). In addition, there is evidence that smartphone overuse impairs cognition *via* its negative influences on mood and sleep (24).

In China, there have been a few studies examining the relationship between smartphone use and sleep problems in university students. Similar associations of sleep problems with MPA, long-term smartphone use, smartphone use before going to bed, and sleeping near the phone, have been reported (25–30). A prospective cohort study of Chinese college students has shown that long-time mobile phone use predicts sleep disturbances, and there is a bidirectional relationship between the duration of phone use and various sleep outcomes (25). Thus, results from both international and Chinese studies suggest that both some characteristics of smartphone use (i.e., duration of phone use, bedtime smartphone use, et al.) and MPA are involved in the etiology of the sleep problems of university students. However, it remains unknown which type of smartphone use variables (i.e., long duration of phone use versus MPA) is the strongest predictor of sleep problems, because these prior studies seldom examined the association of sleep problems with the two types of variables simultaneously. The relative contributions of the two groups of variables to sleep problems are potentially important, because these data may inform the level of intervention intensity required (i.e., health education vs. psychotherapy) if we want to address the sleep problems of university students *via* reducing problematic smartphone use.

Changsha is a major municipality in central-south China with a total population of 7,918,100, of which 672,000 are university students from its 51 universities. Due to societal concern about the misuse of smartphones by university students, our research team focuses on the prevention of MPA and its association with health problems in university campuses. To facilitate the design of a large-scale municipality-wide study on smartphone use and sleep health in university students of Changsha, China, a preliminary study was carried out to investigate the prevalence of poor sleep quality in a sample of Chinese college students, with a particular focus on its associated smartphone use factors. We hypothesized that long time phone use, high expense, and MPA would be negatively correlated with sleep quality in Chinese college students.

## Materials and Methods

### Subjects

This cross-sectional study was conducted in May 2016. By using cluster sampling, all college students of the ten classes of the Department of Pharmacy, Changsha Health Vocational College, were invited to participate in this study. Students who were unwilling to join the study and had severe physical illnesses were excluded.

The study protocol was approved by the Institutional Review Board of Changsha Health Vocational College. Written consent was obtained from all participants, as well as their headteachers and parents, when necessary.

### Procedures and Measures

We used a self-administered questionnaire to collect demographic information and data on psychosocial factors, physical health, smartphone use characteristics, and MPA. Before starting the study, the questionnaire was tested with a small sample of college students, and it was finalized after the pilot study.

Demographic data collected included gender, age, place of origin (rural vs. urban), and self-rated level of family income (good, fair, poor).

Psychosocial factors were depression and social support. In China, the University Personality Inventory (UPI) is the most widely used scale for assessing the mental health of college students (31). It consists of 60 items that assess a wide variety of psychopathological symptoms that an individual may have experienced in the previous year. Nevertheless, the administration time of UPI is a little long, and, therefore, it is not suitable for an epidemiological survey. Importantly, the factor structure of UPI in Chinese college students varies across studies (32–34), making it difficult to explain the test results of UPI. After expert consultation, we decided to use 12 items of the UPI to operationally construct a scale of depressive symptoms (SDS): poor appetite (item 1), feeling pessimistic (item 13), distracted (item 14), or restless (item 23), having suicidal thoughts (item 25), no interest in anything (item 26), a lack of judgment (item 29), good mood (item 35, reverse-scored), or lack of confidence (item 38), and feeling self-abased (item 44), physically exhausted (item 46), and hesitant (item 52). Because these items corresponded to the symptomatology of major depression according to DSM-IV criteria (35), SDS was defined in this manner. To avoid the overlap of the insomnia symptoms of depression and the outcome of our study, sleep quality, item 16 of the UPI (frequent insomnia) was not included in the SDS. All items were answered on a binary (yes/no, rated 1/0) scale. The total score of SDS ranged between 0 and 12, with a higher score denoting more severe depression. In our sample of college students, the internal consistency of SDS was satisfactory (Cronbach's α coefficient: 0.751).

We used two subscales of the Social Support Rating Scale (SSRS) to assess the level of social support: subjective social support and utilization of social support. Developed by Shuiyuan Xiao in 1986, the SSRS has good reliability and validity (36). A higher total score in each subscale represents a higher level of social support (37).

Two questions were used to evaluate physical health: “During the past year, did you often feel that you were in good physical health?” and “During the past year, did you often suffer from headaches?” Respondents who answered “yes” were categorized as having good physical health or headaches.

Smartphone use-related variables included length of smartphone use (years), smartphone charge per month (Yuan), duration of daily smartphone use and mobile internet use (hours), and MPA. MPA was assessed with the validated Chinese Mobile Phone Addiction Index (MPAI), which has 17 items and gauges four dimensions of MPA: inability to control cravings (ICC), feeling anxious and lost (FAL), withdrawal or escapism (WE), and productivity loss (PL) (38, 39). Each item of the MPAI is rated on a five-point Likert scale, from 1=never to 5=always. Higher scores indicate greater severity of MPA.

The primary outcome of the present study, sleep quality, was assessed with the validated Chinese Pittsburgh Sleep Quality Index (PSQI) (40, 41). The PSQI has 18 items,and its total score varies between zero and 21, with a higher score indicating poorer sleep quality. In China, a total score of eight or more is indicative of poor sleep quality (42).

### Statistical Analysis

Respondents were categorized into subgroups according to demographic, psychosocial, physical health, and phone use characteristics. Prevalences of poor sleep quality in the whole sample and in different subgroups of college students were calculated. Chi-square (χ^2^) test and Fisher's exact test were used to compare poor sleepers and normal sleepers between subgroups. Multicollinearity of independent variables was tested prior to the formal analysis. We found a very low degree of multicollinearity among independent variables because the Variance Inflation Factor values of all independent variables ranged from 1.02 to 1.16, substantially below the critical threshold of 10. Multiple logistic regression with a backward stepwise entry of all significant factors in univariate analysis was used to identify factors associated with poor sleep quality. Odds ratio (OR) and 95% confidence intervals (CIs) were generated for each variable. The statistical significance level was set at p < 0.05 (two-sided). SPSS software version 20.0 package was used for all analyses.

## Results

A total of 443 college students were invited to participate in the study, and all agreed. Four students were excluded due to an incomplete questionnaire, so the final sample consisted of 439 completers. The average age of the 439 college students was 18.8 years (standard deviation [SD]: 1.7, range: 15-24), and 86.8% were female. The mean length of smartphone use was 3.9 years (SD: 2.3). Detailed socio-demographic and smartphone use characteristics are displayed in **Table 1**.

**Table 1:** Socio-demographic and smartphone use characteristics of college students and comparison between poor sleepers and normal sleepers by variable.

Variable		No.	Poor sleepers	Normal sleepers	χ^2^	P
Gender	Male	58	10	48		
	Female	381	33	348	4.194	0.041
Age	≤19 years	274	22	252		
	≥20 years	165	21	144	5.573	0.109
Place of origin	Rural	246	27	219		
	Urban	193	16	177	0.883	0.347
Self-rated family economic status	Good	26	3	23		
	Fair	256	21	235		
	Poor	157	19	138	1.984^#^	0.364
Subjective social support*	≤9	227	25	202		
	>9	212	18	194	0.790	0.374
Utilization of social support*	≤8	288	32	256		
	>8	151	11	140	1.641	0.200
Depressive symptoms*	≤2	220	13	207		
	>2	219	30	189	7.537	0.006
Good physical health	Yes	381	31	350		
	No	58	12	46	8.978	0.003
Headaches	Yes	92	18	74		
	No	347	25	322	12.575	<0.001
Characteristics of phone use						
Years of smartphone use*	≤4	267	17	250		
	>4	172	26	146	9.063	0.003
Monthly smartphone charge (Yuan)*	≤50	236	17	219		
	>50	203	26	177	3.880	0.049
Hours of daily smartphone use*	≤5	249	16	233		
	>5	190	27	163	7.392	0.007
Hours of daily mobile internet use*	≤5	260	12	248		
	>5	179	31	148	19.362	< 0.001
Mobile phone addiction index						
Inability to control cravings*	≤14	239	15	224		
	>14	200	28	172	7.352	0.007
Feeling anxious and lost*	≤7	258	18	240		
	>7	181	25	156	5.625	0.018
Withdrawal or escapism*	≤7	259	25	234		
	>7	180	18	162	0.015	0.904
Productivity loss*	≤5	231	13	218		
	>5	208	30	178	9.583	0.002

*All continuous variables were dichotomized at the median value.

^#^Fisher's exact test.

The mean PSQI score was 4.5 (SD: 2.3). A total of 43 students (9.8%) had poor sleep quality (PSQI > 7). Results from Chi-square tests and Fisher's exact test (**Table 1**) show that poor sleepers were more likely to be male students (P=0.041), not to have good physical health (P=0.003), to suffer from headaches (P < 0.001), to have more severe depressive symptoms (as indicated by a score of two or more, P=0.006), to have been using a smartphone for more than four years (P=0.003), to pay a monthly phone charge of 50 Yuan or more (P=0.049), to use the phone for more than five hours per day (P=0.007), to use mobile internet for more than five hours per day (P < 0.001), and to have more severe MPA in terms of ICC (P=0.007), FAL (P=0.018), and PL (P=0.002).

Multivariable logistic regression analysis (**Table 2**) show that poor sleep quality in college students was significantly associated with male gender (OR: 2.80, P: 0.022), not having good physical health (OR: 2.61, P: 0.020), headaches (OR: 2.47, P: 0.014), more severe depressive symptoms (OR: 2.17, P: 0.049), > four years of smartphone use (OR: 3.38, P: 0.001), > five hours of daily smartphone use (OR: 2.19, P: 0.049), and more severe ICC (OR: 2.04, P: 0.040).

**Table 2:** Multivariable logistic regression of factors associated with poor sleep quality.

Variable	Risk level	Reference level	P	OR(95%CI)
Gender	Male	Female	0.022	2.80(1.16,6.71)
Depressive symptoms*	>2	≤2	0.049	2.17(1.01,4.68)
Having good physical health	No	Yes	0.020	2.61(1.16,5.88)
Headaches	Yes	No	0.014	2.47(1.20,5.07)
Years of smartphone use*	>4	≤4	0.001	3.38(1.67,6.87)
Hours of daily smartphone use*	>5	≤5	0.049	2.19(1.04,4.63)
Inability to control cravings*	>14	≤14	0.040	2.04(1.01,4.14)

*All continuous variables were dichotomized at the median value.

## Discussion

According to the statistics provided by the China Internet Network Information Center (CNNIC), by June 2019, the number of internet users in China was 854 million, and 847 million (99.1%) used mobile phones to access the internet. Of the total internet users, 41.5% were 10–29 years old, and 26% were students (the largest group) (43). Therefore, Chinese adolescents and young adults such as college students are the most vulnerable group to problematic smartphone use. To the best of our knowledge, our study is one of the very few to examine associations of poor sleep quality with smartphone use characteristics and MPA. We found that nearly one-tenth (9.8%) of the Chinese college students reported poor sleep quality and that poor sleep quality was significantly associated with male gender, not having good physical health, headaches, more severe depressive symptoms, >four years of smartphone use, >five hours of daily smartphone use, and more severe ICC of MPA.

The 9.8% prevalence of poor sleep quality revealed in our sample of college students is exactly the same as that of college students in Anhui, a central province in China (44) but lower than that of Chinese university students (25.7%) and the general population (15.0%) reported by two meta-analytic reviews (3, 4). The lower rate of poor sleep quality in this study might be related to the lower level of academic stress in college students of a health vocational college compared to college students of general universities. However, due to differences in sample characteristics (i.e., percentage of male students) and assessments of sleep problem (i.e., PSQI vs. Athens Insomnia Scale) across existing studies, the relatively low prevalence of poor sleep quality does not necessarily mean a low risk of sleep problems in our sample of college students. Considering the many negative health effects associated with sleep problems and the very large number of Chinese college students, this 9.8% prevalence figure suggests that the sleep problems of college students still deserve preventive and clinical attention.

Overall, the significant associations between poor sleep quality and poor physical health (as indicated by not having good physical health and headache) and depression are consistent with prior studies (45–47). These could be explained by the complex reciprocal relationships between sleep disorders and pain, chronic conditions, and depression (46, 48–50). For example, poor physical and mental health would cause insomnia, and insomnia would exacerbate physical and mental health problems.

Numerous studies have confirmed the female predisposition to sleep problems in various populations, including college students, young adults, and adults (51–55), but we found a significantly elevated risk of poor sleep quality in male compared to female students. This unexpected phenomenon might be related to some unknown male-specific stressors, because, in most Chinese health vocational colleges, male students are the minority group, usually accounting for less than 5% of the total students. Nevertheless, since the sample size of male students is small (n=58), further investigation is warranted to validate this finding.

Our findings on the significant relationships between poor sleep quality and excessive smartphone use (as indicated by more years of smartphone use and more hours of daily smartphone use) and more MPA-associated ICC are generally in line with existing international and Chinese studies, despite the different measures of smartphone overuse and MPA used (17–23, 25–30). The difference between this study and other studies is that excessive smartphone use and MPA simultaneously contributed to the poor sleep quality of college students. Although the long-term effect of smartphone use on health remains unclear, the identified association between more years of smartphone use and poor sleep quality in our study might suggest a cumulative adverse effect of smartphone use on sleep.

Several mechanisms have been proposed to explain the negative effect of smartphone use on sleep. First, long-time phone use directly reduces the user's time spent on sleep, in particular bedtime smartphone use, resulting in insufficient sleep. Second, some phone users prefer to browse mobile websites before bed. Inappropriate content on these websites may make the users feel tension and excitement, leading to difficulties in initiating sleep. Third, excessive smartphone use may affect sleep *via* some physiological and psychological pathways (27, 56). For example, exposure to the light of smartphone screens and radiation from the phone during bedtime could influence the onset time and secretion of melatonin, which in turn causes sleep-wake rhythm disorders (25, 57). Fourth, morphological evidence from an fMRI study has confirmed alterations in gray matter volume and white matter integrity in college students with MPA (58); we speculate that MPA would affect sleep *via* several neurobiological pathways, which need to be further understood.

As a preliminary study, some limitations are unavoidable. First, this is a cross-sectional study, so the significant associations of poor sleep with poor physical health, depression, unhealthy smartphone use, and MPA found are not, strictly speaking, causal relationships. The influences of these factors on sleep need to be examined by prospective longitudinal studies. Second, our sample of college students was recruited from one health vocational college only, so the sample representativeness is very limited. Caution is needed when generalizing our findings to students of other colleges and universities. Third, although the internal consistency of our measure of depression was satisfactory and its items were believed to mirror depressive symptoms, empirical data on its validity were still unavailable in this study. Studies using common depression scales are warranted to examine the sleep–smartphone use associations. Fourth, our assessment of perceived physical health was based on only a single question, which may be unable to capture all dimensions of physical well-being. Fifth, there are no clinical diagnoses of mobile phone addiction, and the results from MPA assessment can only indicate the severity of phone addiction. Sixth, there are some other limitations, such as inadequate controlling of other variables associated with poor sleep, such as anxiety, and no assessment of the contents browsed on the phone before falling asleep, which could influence sleep quality.

## Conclusions

In summary, in this sample of Chinese college students from a health vocational college in Changsha, China, one out of every ten have poor sleep quality, and poor sleep quality is significantly associated with poor physical health, depression, excessive smartphone use, and MPA. Given the common use of smartphones in Chinese colleges and the association of this use with sleep problems, administrators and health workers at Chinese colleges should pay more attention in terms of research and interventions to problematic smartphone use among students. Further investigation is justified into whether reducing smartphone use and MPA can improve the sleep quality of Chinese college students. Nevertheless, because of the limited sample representativeness and cross-sectional design of this study, large-scale prospective representative studies are warranted to confirm these associations.

## Data Availability Statement

All datasets generated for this study are included in the article/supplementary material.

## Ethics Statement

The studies involving human participants were reviewed and approved by the Institutional Review Board of Changsha Health Vocational College. The patients/participants provided their written informed consent to participate in this study.

## Author Contributions

All authors made substantial contributions to this study. QH and HC conceptualized and designed the research. XZ, JQ, and SL prepared the assessment tools. TS, ZL, and XC performed the experiments. YC and YL undertook the statistical analysis. QH and SH wrote the first draft of the manuscript and contributed to the final manuscript. All authors critically reviewed content and approved the final version for publication.

## Funding

This work was supported by the National Natural Science Foundation of China (81971249), the National Basic Research Program of China (2015CB553504), and the National Research Program of China (2016YFC0800908-Z02).

## Conflict of Interest

The authors declare that the research was conducted in the absence of any commercial or financial relationships that could be construed as a potential conflict of interest.
